# Entropy-Based Measure of Statistical Complexity of a Game Strategy

**DOI:** 10.3390/e22040470

**Published:** 2020-04-20

**Authors:** Fryderyk Falniowski

**Affiliations:** Department of Mathematics, Cracow University of Economics, Rakowicka 27, 31-510 Kraków, Poland; falniowf@uek.krakow.pl

**Keywords:** entropy-based measure, complexity of a strategy, predictability of a strategy

## Abstract

In this note, we introduce excess strategic entropy—an entropy-based measure of complexity of the strategy. It measures complexity and predictability of the (mixed) strategy of a player. We show and discuss properties of this measure and its possible applications.

## 1. Introduction

Many social (economic, political, etc.) interactions have been modeled as formal games. In particular, repeated games play the central role in models of the long-term competition in economic theory and modeling interactions which are repeated frequently [1,2]. In such games, a strategy is a set of history-contingent plans of action. Each plan of action (play) is an infinite sequence of pairs (for two-player games) or *n*-tuples (in the *n*-player game) of strategies in each stage game. In every stage game, each player uses a mixed strategy (in order to avoid possible confusion, we point out that, as is standard in the literature, we consider mixed strategies so long as their support lies in the set of feasible pure strategies; a possible interpretation of mixed strategies in games in general is that they are distributions of pure strategies in a population of potential players). Generally, in the literature, it is assumed that players can carry out any strategy in a specified strategy set, should they choose to play it. While this latter assumption may seem innocuous in a model where few strategies are available to each player, it may be criticized as being unrealistically rational in more complex models where a theoretical definition of strategy leads to a strategy set that contains a large number of choices, many of which are impractically complex. This is usually the case in repeated games—at every stage, players are playing a history (in-)dependent game and therefore the set of all strategies can be huge. Thus, it is reasonable to consider the complexity of the strategy (the idea that the assumption of fully, or unboundedly, rational players is unrealistic is not new; see, e.g., [3]). In addition, putting the complexity problem in the wider context, one of the main benchmark results in the theory of repeated games is the folk theorem. It demonstrates that any individually rational and feasible payoff vector of a two-player normal form game is a perfect equilibrium outcome of the repeated game, when the discount factor is sufficiently near one [4]. This multiplicity of equilibria has led some researchers, both theoretical and applied, to restrict attention to certain equilibria. Along with other restrictions, the restriction to certain simple strategies, such as the grim trigger strategy, is commonly used. Moreover, these types of equilibrium selection arguments are not restricted to repeated game. Such restrictions are the norm rather than the exception in dynamic models in economics. Most analyses of dynamic macro models, search models, and random matching models restrict attention to equilibria in which agents use history-independent strategies. This is also true for many models in dynamic games, including stochastic games and multi-person bargaining.

In general, restricting the set of feasible strategies having regard to complexity of the strategy seems to be particularly relevant. A popular approach to the problem of complexity of strategies is to assume bounded rationality of an agent. There have been many attempts to model feasible (implementable) sets of strategies that reflect some aspects of the bounded rationality of players. Finite automata, bounded recall, and Turing machines are a few of the approaches taken. These models are useful because they provide us with quantitative measures of complexity of strategies, e.g., number of states of automata and the length of recall. This approach, used e.g., in [5,6], describes the complexity of the strategy from the point of view of an individual who wants to implement the strategy, and is based on the idea similar to Kolmogorov–Chaitin complexity of a channel in the context of information theory. However, the complexity of the strategy comes not only from the fact of how complicated it is to implement. The complex, structured strategy is the strategy that is difficult to predict. The player may face the problem of choosing the best strategy against the strategy of the opponent, which he learns during the repeated game. The more structured the strategy is, the more difficult it is to predict what the opponent will do, and the more difficult it is to find the best response strategy. Therefore, there is a necessity to consider measures that would help us to detect more or less structured strategies.

In the seminal article, Neyman and Okada introduced entropy-based measures of uncertainty for mixed strategies of repeated games—strategic entropy and strategic entropy rate [7]. They studied repeated two-player zero-sum games in which a player, say Player 1, with a restricted set of strategies plays against an unrestricted player, Player 2. Concerning the values of such games, two questions arise: “What is the number of repetitions needed for Player 2 to take advantage of Player’ 1’s restriction?” and “How long can Player 1 protect himself against an unrestricted Player 2?” These questions can be posed in the language of asymptotic behavior of the value of the repeated game: “What is the relationship between the number of repetitions and the unpredictability bound so that, as they tend to infinity, either (a) Player 2 can hold Player 1’s payoff down to his one-shot game max-min value in pure actions or (b) Player 1 can still secure the value of the one-shot game?” They imposed a restriction directly on mixed strategies. To this end, they employed entropy as a measure of uncertainty of the mixed strategy relative to the other player’s strategy. The strategic entropy rate of a mixed strategy is the maximal entropy rate of the play with respect to the other player’s strategy. Thus, it is the maximal uncertainty of the play that the other player faces against (mixed) strategy. Of course, their entropy concept is not a measure of the strategic complexity in the way that the size of the automaton or the length of recall was intended to be but captures an abstract informational feature common in bounded recall restrictions, and thereby serves as a useful tool to analyze them.

To provide a proper and more precise insight into the structure and complexity of strategy, we propose taking the next step—looking at convergence rates of the entropy. It is commonly agreed that a slow entropy convergence rate is a sign of complex structure of a process [8]. Obviously, a more structured strategy is more difficult to predict. Therefore, strategies with a slow convergence rate are less predictable. Hence, restricting the set of feasible strategies to the set of those with bounded unpredictability can give more precise insight on the theory of long-term competition.

We propose to use the concept of excess entropy, which measures an entropy convergence rate. It can result in better understanding of strategies played—measuring not only randomness of the strategy but its structure, regularity, and predictability as well. To this end, we will construct a measure based on the quantity widely used in information theory and physics.

Structure and correlation are not completely independent of randomness. It is generally agreed that both maximally random and perfectly ordered systems possess no structure [9]. Nevertheless, at a given level of randomness away from these extremes, there can be an enormously wide range of differently structured processes. There are many ad hoc methods for detecting structure, but none are as widely applicable as entropy is for indicating randomness. The quantities that have been proposed as general structural measures are often referred to as *complexity measures*. To reduce confusion, it has become convenient to refer to them instead as *statistical* complexity measures. In doing so, they are immediately distinguished from deterministic complexities. In contrast, statistical complexity measures discount for randomness, and so provide a measure of the regularities present in an object above and beyond pure randomness.

In 1983, Grassberger and Procaccia in their seminal paper [10] introduced the measure of complexity—*excess entropy* (they called it the *effective complexity measure*) to study complexity of chaotic signal. It made a huge impact in particular on statistical mechanics and information theory (*Google Scholar* records over 1600 citations of this article), and in economics as well [11]. The most precise and consistent approach to this measure can be found in the article by Crutchfield and Feldman [8]. The main idea is to look carefully at the manner in which block entropy H(n), n∈N, converges to its asymptotic form. Let us consider infinite sequences defined over the alphabet A. We define the *n*-block entropy as

$$H\left(n\right)=-\sum_{s^{n}\in A^{n}}\mu \left(s^{n}\right)\log\mu \left(s^{n}\right),$$

where $s^{n}$ are blocks of length *n* and μ is a probability distribution. The average (per-symbol) uncertainty is given by

$$h=\lim_{n\to \infty }\frac{H(n)}{n}=\lim_{n\to \infty }\left(H\left(n+1\right)-H\left(n\right)\right).$$

After defining these quantities, the excess entropy can be introduced as

$$E=\sum_{n=1}^{\infty }\left(H\left(n+1\right)-H\left(n\right)-h\right).$$

The excess entropy has a number of different interpretations and goes also by a variety of different names (also *stored information*, *effective measure complexity*, *Grassberger’s complexity*, and *predictive information*). Among others, it measures the excess randomness of the system, and therefore tells us how much additional information must be gained about the sequences in order to reveal the actual per-symbol uncertainty *h*. Thus, it can be interpreted as a measure of statistical complexity.

The aim of this note is to incorporate the concept of excess entropy into the game theory setting. It can be used to answer the question how fast the strategic entropy of the (first) *n* stage games of the repeated game converges to the limit that is to the (strategic) entropy rate of the strategy. To this aim, we propose an analogue of excess entropy, which we will call excess strategic entropy (shortly: *excess s-entropy*). We will use it to measure statistical complexity and unpredictability of strategies. Natural interpretations of this measure impose its applicability e.g., in the case of bounded recall and reputation models. Undertaken research and obtained results fit into the growing field of applications of entropy-based concepts in economics, and game theory in particular (see [12,13,14,15,16,17] and references therein).

## 2. Results

### 2.1. Formal Description of the Game

This section is intended to make the reader familiar with basic concepts of game theory. For the clarity of presentation, we consider a two-player zero-sum games. A *play* of a repeated game is an infinite sequence $\omega ={\left(\omega_{k}\right)}_{k=1}^{\infty }$, where $\omega_{k}=\left(a_{k},b_{k}\right)\in A\times B$. We denote the set of all plays by Ω, i.e., $\Omega ={\left(A\times B\right)}^{N}$.

Two plays $\omega ={\left(\omega_{k}\right)}_{k=1}^{\infty }$ and $\omega^{\prime }={\left(\omega_{k}^{\prime }\right)}_{k=1}^{\infty }$ are said to be *n-equivalent* if $\omega_{k}=\omega_{k}^{\prime }$ for k=1,…,n. The *n*-equivalence is clearly an equivalence relation on Ω. Denote by $Q_{n}$ the finite partition of Ω into *n*-equivalence classes. Each *n*-equivalence class of plays is called an *n-history*, and it represents the information available to the players at the end of stage *n*.

We denote by $\Sigma_{n}$ the algebra on Ω generated by $Q_{n}$. Set $\Sigma_{0}=\left\{\emptyset ,\Omega \right\}$. Clearly, $\Sigma_{n-1}\subset \Sigma_{n}$ for every n∈N. The σ-algebra generated by ${⋃}_{n\geq 0}\Sigma_{n}$ is denoted by $\Sigma_{\infty }$.

$S_{n}$ and $T_{n}$ denote the sets of measurable mappings from $\left(\Omega ,\Sigma_{n-1}\right)$ to *A* and *B*, respectively. Each element of $S_{n}$ and $T_{n}$ represents a ’strategy at stage *n*’. For each $s_{n}\in S_{n}$ (and similarly for $t_{n}\in T_{n}$), since $s_{n}\left(\omega \right)$ depends only on the first *n* coordinates of ω, we sometimes write $s_{n}\left(\omega_{1},\ldots ,\omega_{n-1}\right)$. A *pure strategy* of Player 1 (resp. 2) is a sequence $s=(s_{n})$ with $s_{n}\in S_{n}$ ($t=(t_{n})$ with $t_{n}\in T_{n}$, respectively). Thus, the sets of pure strategies of the two players are $S=\times_{n\geq 1}S_{n}$ and $T=\times_{n\geq 1}T_{n}$. We consider *S* and *T* to be endowed with the product topologies with the discrete topology on each factor. S and T denote the Borel σ-algebra of *S* and *T*, respectively. A *mixed strategy* of Player 1 (resp. 2) is then a probability on (S,S) (resp. (T,T)).

Every pair of pure strategies (s,t) induces a play $\omega ={\left(\omega_{k}\right)}_{k=1}^{\infty }\in \Omega$, where $\omega_{k}$ is defined inductively

$$\omega_{k}=\left(a_{k},b_{k}\right)=\left\{\begin{array}{cc} \left(s_{1},t_{1}\right) & \mathrm{for}\;k=1\\ \left(s_{k}\left(\omega_{1},\ldots ,\omega_{k-1}\right),t_{k}\left(\omega_{1},\ldots ,\omega_{k-1}\right)\right) & \mathrm{for}\;k>1 \end{array}\right..$$

Note that $s_{1}$ and $t_{1}$, being $\Sigma_{0}$-measurable, are constant everywhere. Accordingly, every pair (σ,τ)∈Δ(S)×Δ(T) induces a probability $P_{\sigma ,\tau }$ on $\left(\Omega ,\Sigma_{\infty }\right)$. Equivalently, (σ,τ) induces a sequence of random actions, or a *random play*$\left(X_{k}\right)$, where $X_{k}=\left(a_{k},b_{k}\right)$ is an (A×B)-valued random variable.

**Remark** **1.** *To simplify discussion, one can identify A (and B) with the set of non-negative integers A={0,1,…,cardA} (and B={0,1,…,cardB}, respectively). Then the space *Ω* can be represented as the full shift $\left(A^{N},\tau \right)$, where $A^{N}$ is a set of all one-sided infinite sequences over alphabet A, and $\tau :A^{N}\mapsto A^{N}$ is a shift transformation ${\left(\tau x\right)}_{n}=x_{n+1}$. If at every stage every action is accessible, then *Ω* is the set of all one-sided sequences on A. On the other hand, if only some of actions are accessible for Player 1 or Player 2, then the space of possible histories is a subshift of *Ω*. Moreover, the partition imposed by n-equivalence classes is the partition into cylinders; see Section 2.2.2.*

### 2.2. Basic Concepts

#### 2.2.1. Information Theoretic Concepts

In this subsection, we recall well-known quantities and facts from information theory. Let Γ be a finite set and *X* be a random variable which takes values in Γ, and whose distribution is p∈Δ(Γ), i.e., p(γ)=Prob(X=γ) for each γ∈Γ. To simplify the notation, we define a function η:[0,∞)↦R by

$$\eta \left(x\right)=\left\{\begin{array}{cc} -x\log x & \mathrm{for}\;x>0,\\ 0 & x=0. \end{array}\right.$$

**Definition** **1.** *The entropy H(X) of X defined as the expected value of the information is equal to*

$$H\left(X\right)=E\left(\log p^{-1}\right)=\sum_{\gamma \in \Gamma }\eta \left(p\left(\gamma \right)\right).$$

The notion of entropy can be naturally extended to an arbitrary finite dimensional vector of random variables or probability distributions.

**Definition** **2.** *The conditional entropy $H(X_{2}|X_{1})$ of $X_{2}$ given $X_{1}$ is defined as*

$$H\left(X_{2}|X_{1}\right)=\sum_{\gamma \in \Gamma }p\left(\gamma \right)H_{p(\gamma )}\left(X_{2}\right),$$

**Lemma** **1** (see [18]). *H(Y|X)=H(X,Y)-H(X) and more generally*

$$H\left(X_{1},\ldots ,X_{n}\right)=H\left(X_{1}\right)+\sum_{i=2}^{n-1}H\left(X_{i}|X_{1},\ldots X_{i-1}\right)\leq \sum_{i=1}^{n}H\left(X_{i}\right).$$

**Definition** **3.** *Let $\left(X_{n}\right)$ be a stochastic process, where each $X_{n}$ takes values in a finite set *Γ*. The entropy rate of the process $\left(X_{n}\right)$ is defined as*

(1) $$h\left(\left(X_{n}\right)\right)=\underset{k\to \infty }{lim sup}\frac{1}{k}H\left(X_{1},\ldots ,X_{k}\right).$$

#### 2.2.2. Stationary Processes and Symbolic Dynamics

Stationary processes (and induced by them stationary strategies) will be an important tool in our considerations. In this section, we recall the concept of a stationary process and show the connection between stationary process, and existence of a shift-invariant measure (an in-depth discussion of these connections can be found in [19]).

We say that process $\left(X_{n}\right)$ is stationary, if the joint distributions do not depend on the choice of time origin, that is,

(2) $$Prob\left(X_{i}=a_{i},\;m\leq i\leq n\right)=Prob\left(X_{i+1}=a_{i},\;m\leq i\leq n\right)$$

for all m,n∈N and $a=\left(a_{1},a_{2},\ldots \right)\in A^{N}$. The statement that a process $\left(X_{n}\right)$ on the finite alphabet A is stationary, which simply translates into the statement that the Kolmogorov measure introduced on cylinder sets

$$\left[a_{m}^{n}\right]=\left\{x\in A^{N}:x_{i}=a_{i},m\leq i\leq n\right\}$$

by

(3) $$\mu \left(\left[a_{1}^{n}\right]\right)=\mu \left(\left\{x:x_{i}=a_{i},\;1\leq i\leq n\right\}\right)=Prob\left(X_{i}=a_{i},\;1\leq i\leq n\right)$$

is invariant under the shift transformation $\tau :A^{N}\mapsto A^{N}$, that is,

$$\mu \left(B\right)=\mu \left(\tau^{-1}B\right)$$

for any measurable set *B*.

In this note, we will focus our attention on stationary strategies.

**Definition** **4.** *A strategy profile (σ,t)∈Δ(S)×T is called stationary (strategy σ is stationary with respect to t) if a random play $\left(X_{n}\right)$ induced by (σ,t) is stationary.*

For stationary processes, the upper limit in Formula (1) can be replaced by the limit. In the next section (Lemma 3), we will show a similar fact for stationary strategies.

### 2.3. Strategic Entropy Rate

#### 2.3.1. Derivation of the Strategic Entropy Rate

We define the strategic entropy rate of the strategy σ∈Δ(S), given strategy t∈T, following construction proposed in [7]. It is a measure of average degree of uncertainty which Player 2 suffers playing the pure strategy *t* if Player 1 plays a mixed strategy σ.

For each n∈N, we define a function $H_{n}\left(\cdot ,\cdot \right):\Delta \left(S\right)\times T\mapsto R$ as follows. For a given (σ,t)∈Δ(S)×T, let $\left(X_{k}\right)$ be the random play induced by (σ,t). Then, $H_{n}\left(\sigma ,t\right)$ is defined as the entropy of this random play up to stage *n*, that is,

$$H_{n}\left(\sigma ,t\right)=H\left(X_{1},\ldots ,X_{n}\right)=\sum_{C\in Q_{n}}\eta \left(P_{\sigma ,t}\left(C\right)\right).$$

Recall that $Q_{n}$ is the partition of Ω with respect to actions in the first *n* stages. Therefore, $H_{n}\left(\sigma ,t\right)$ is the uncertainty about the play up to the stage *n* that Player 2 faces when Player 1 uses σ and Player 2 uses *t*. The dual interpretation is that it is the amount of information on the play of the game, which Player 2 can obtain using *t* when Player 1 uses σ. Properties of $H_{n}$ are listed in the following lemma, and its proof can be found in [7]:

**Lemma** **2.**

*1*. *$H_{n}\left(\cdot ,\cdot \right)$ is continuous on Δ(S)×T.*
*2*. *For each t∈T, $H_{n}\left(\cdot ,t\right)$ is concave on Δ(S) and constant on each equivalence class of Δ(S),*
*3*. *For each σ∈Δ(S), $H_{n}\left(\sigma ,\cdot \right)$ is constant on each n-equivalence class of T,*
*4*. *$H_{n}\left(\sigma ,t\right)\in \left[0,n\log\mathrm{card}A\right]$ for every (σ,t)∈Δ(S)×T.*

*Moreover, if $\left(X_{k}\right)$ is the random play induced by (σ,t), then*

$$H_{n}\left(\sigma ,t\right)=H\left(X_{1}\right)+\sum_{k=2}^{n}H\left(X_{k}|X_{1},\ldots ,X_{k-1}\right).$$

Now, we can define the strategic entropy rate.

**Definition** **5.** *If $\left(X_{n}\right)$ is the random play induced by (σ,t)∈Δ(S)×T, then the entropy rate of the strategy σ with respect to the strategy t is*

(4) $$h\left(\sigma ,t\right)=\underset{n\to \infty }{lim sup}\frac{1}{n}H_{n}\left(\sigma ,t\right)=\underset{n\to \infty }{lim sup}\frac{1}{n}H\left(X_{1},\ldots ,X_{n}\right)$$

*and the strategic entropy rate of the strategy σ is the supremum of h(σ,t) over t∈T:*

(5) $$h\left(\sigma \right)=\underset{t\in T}{\sup}h\left(\sigma ,t\right).$$

In general, the limit of $H_{n}\left(\sigma ,t\right)/n$ doesn’t have to exist. However, in the next section, we will show that, for a large class of strategies, it does.

#### 2.3.2. Limits for Stationary Strategies

We start this section with a discussion on the existence of the limit in Formula (4) for stationary strategies.

**Lemma** **3.** *Sequences $h_{n}\left(\sigma ,t\right)=\frac{1}{n}H_{n}\left(\sigma ,t\right)$ and $g_{n}\left(\sigma ,t\right)=\left(H_{n+1}\left(\sigma ,t\right)-H_{n}\left(\sigma ,t\right)\right)$ are non-negative and*

$$\underset{n\to \infty }{lim sup}g_{n}\left(\sigma ,t\right)=\underset{n\to \infty }{lim sup}h_{n}\left(\sigma ,t\right)=h\left(\sigma ,t\right).$$

*If additionally σ is stationary, then they are decreasing and have a common limit.*

**Proof.**  For any process, we have that they are non-negative since

$$\begin{array}{cc} H_{n+1}\left(\sigma ,t\right) & =H\left(X_{1},\ldots ,X_{n+1}\right)=H\left(X_{1},\ldots ,X_{n}\right)+H\left(X_{n+1}|X_{1},\ldots ,X_{n}\right)\\ & \geq H\left(X_{1},\ldots ,X_{n}\right)=H_{n}\left(\sigma ,t\right). \end{array}$$

Moreover, because $h_{n}\left(\sigma ,t\right)$ is equal to the Cesáro average of $g_{n}\left(\sigma ,t\right),$ we obtain that

(6) $$h\left(\sigma ,t\right)=\underset{n\to \infty }{lim sup}g_{n}\left(\sigma ,t\right)=\underset{n\to \infty }{lim sup}h_{n}\left(\sigma ,t\right).$$

From now on, we assume that $\left(X_{n}\right)$ is stationary. Therefore, we can look at the Kolmogorov measure μ (see (3)). To simplify notation, let

$$p_{\iota }=\mu \left(\left\{x:x_{j}=a_{i_{j}},\;1\leq j\leq n\right\}\right)$$

for $\iota =\left\{i_{1},\ldots ,i_{n}\right\}\in A^{n}$. Then, from invariance of the measure μ and strong subadditivity of Shannon entropy [20], we obtain

$$\begin{array}{ccc} H_{n+2}\left(\sigma ,t\right)-H_{n+1}\left(\sigma ,t\right) & = & \sum_{i=1}^{\mathrm{card}A}\sum_{\iota \in A^{n}}\sum_{j=1}^{\mathrm{card}A}\eta \left(p_{i\iota j}\right)-\sum_{\iota \in A^{n}}\sum_{i=1}^{\mathrm{card}A}\eta \left(p_{i\iota }\right)\\ & = & \sum_{i=1}^{\mathrm{card}A}\sum_{\iota \in A^{n}}\sum_{j=1}^{\mathrm{card}A}\eta \left(p_{i\iota j}\right)-\sum_{\iota \in A^{n}}\sum_{i=1}^{\mathrm{card}A}\eta \left(\sum_{j=1}^{\mathrm{card}A}p_{i\iota j}\right)\\ & \leq & \sum_{\iota \in A^{n}}\sum_{j=1}^{\mathrm{card}A}\eta \left(\sum_{i=1}^{k}p_{i\iota j}\right)-\sum_{\iota \in A^{n}}\eta \left(\sum_{i=1}^{\mathrm{card}A}\sum_{j=1}^{\mathrm{card}A}p_{i\iota j}\right)\\ & = & \sum_{\iota \in A^{n}}\sum_{j=1}^{\mathrm{card}A}\eta \left(p_{\iota j}\right)-\sum_{\iota \in A^{n}}\eta \left(p_{\iota }\right)\\ & = & H_{n+1}\left(\sigma ,t\right)-H_{n}\left(\sigma ,t\right). \end{array}$$

Therefore, the sequence $\left(g_{n}\left(\sigma ,t\right)\right)$ is non-negative and decreasing; thus, it has a limit. □

**Corollary** **1.** *If (σ,t)∈Δ(S)×T is stationary, then $h\left(\sigma ,t\right)=\lim_{n\to \infty }\frac{1}{n}H_{n}\left(\sigma ,t\right)$.*

Naturally, we can ask whether we can consider limits instead of the upper limits in Formula (6). Unfortunately, in general, neither $h_{n}\left(\sigma ,t\right)$ (see discussion after Definition 5) nor $g_{n}\left(\sigma ,t\right)$ (see Example 1) have to possess the limit.

**Example** **1.** *Let A={0,1} and let the strategy of σ be the following:*

- *at every even stage, Player 1 chooses 1;*
- *at every stage 4n+1, Player 1 chooses 0 and 1 with equal probability;*
- *at every stage 4n+3, Player 1 chooses 0 with the probability 1/4 and 1 with probability 3/4.*

*Then,*

$$g_{n}\left(\sigma ,t\right)=H_{n+1}\left(\sigma ,t\right)-H_{n}\left(\sigma ,t\right)=H\left(X_{n+1}|X_{1},\ldots ,X_{n}\right).$$

*For every k∈N, we have that*

$$H\left(X_{k}|X_{1},\ldots X_{k-1}\right)=\left\{\begin{array}{cc} 0, & \mathrm{if}\;k\;\mathrm{is}\;\mathrm{even},\\ \log2, & \mathrm{if}\;k=4n+1,\\ -\frac{1}{4}\log\frac{1}{4}-\frac{3}{4}\log\frac{3}{4}, & \mathrm{if}\;k=4n+3. \end{array}\right.$$

*Thus, $g_{n}$ doesn’t have the limit.*

We can see that the assumption that the strategy is stationary gives a nice theory of the strategic entropy (and as we will see in the next section—excess *s*-entropy). One may ask if the assumption of stationarity isn’t too restrictive. Obviously using only stationary strategies may seem to be quite restrictive, but we know that, for many types of games, there exists an optimal stationary strategy [21,22]. Such strategy can be used widely because of its simplicity and optimality.

### 2.4. Excess Strategic Entropy

#### 2.4.1. Excess *s*-Entropy

Now, we are able to introduce the fundamental tool of this note.

**Definition** **6.** *The excess strategic entropy of the strategy σ∈Δ(S) with respect to the strategy t∈T (shortly the excess s-entropy of (σ,t)) is defined as*

$$E\left(\sigma ,t\right)=\sum_{n=0}^{\infty }\left(g_{n}\left(\sigma ,t\right)-h\left(\sigma ,t\right)\right).$$

*We define the excess strategic entropy of the strategy σ∈Δ(S) (excess s-entropy of σ) as*

$$E\left(\sigma \right)=\underset{t\in T}{\sup}E\left(\sigma ,t\right).$$

Excess *s*-entropy is an analogue of the excess entropy concept discussed thoroughly e.g., in [8]. It measures how structured and how complex the strategy is. From the point of view of Player 1, it can be interpreted as a measure of complexity of his choices. On the other hand, from the point of view of Player 2, it can be understood as the measure of predictability of Player 1, that is, how eager Player 1 is to change the (one-shot) strategy. In the following considerations, we show properties of this quantity and calculate excess *s*-entropy of strategies in few games.

**Theorem** **1.** *For any σ∈Δ(S) and t∈T, we have*

$$E\left(\sigma ,t\right)=\underset{n\to \infty }{lim sup}n\cdot h_{n}\left(\sigma ,t\right)=\underset{n\to \infty }{lim sup}n\cdot \left(\frac{1}{n}H_{n}\left(\sigma ,t\right)-h\left(\sigma ,t\right)\right).$$

**Proof.**  Let M∈N. Then,

$$\begin{array}{ccc} \sum_{n=0}^{M-1}g_{n}\left(\sigma ,t\right)-h\left(\sigma ,t\right) & = & H_{M}\left(\sigma ,t\right)-H_{0}\left(\sigma ,t\right)-Mh\left(\sigma ,t\right)\\ & = & H_{M}\left(\sigma ,t\right)-Mh\left(\sigma ,t\right)=M\left(\frac{H_{M}\left(\sigma ,t\right)}{M}-h\left(\sigma ,t\right)\right). \end{array}$$

Taking the upper limit over *M*, we complete the proof. □

Directly from Theorem 1, we obtain the condition for finiteness of excess *s*-entropy.

**Corollary** **2.** *If $h_{n}\left(\sigma ,t\right)∼\frac{1}{n}$ that is*

$$0<\underset{n\to \infty }{lim inf}nh_{n}\left(\sigma ,t\right)\leq \underset{n\to \infty }{lim sup}nh_{n}\left(\sigma ,t\right)<\infty ,$$

*then E(σ,t)∈(0,∞).*

Obviously, having equivalent formulas for excess *s*-entropy, one may ask which of these two is better to use. Both of them are useful. Because $h_{n}$ is a Césaro average of $g_{n}$, it is easy to see that $g_{n}$ converges to the (upper) limit faster than $h_{n}$, so it is better to use it in numerical computations. On the other hand, calculating the upper limit of $\left(n\left(h_{n}-h\right)\right)$ can be easier analytically.

#### 2.4.2. Examples

Now, we give examples of excess *s*-entropy of a strategy for few games.

**Example** **2** (Pure actions). *If σ is a pure strategy, then $H_{n}\left(\sigma ,t\right)=0$ for every n, and thus E(σ)=0.*

**Example** **3** (Independent actions). *Let $\sigma =(\sigma_{k})$ be a sequence of independent mixed actions, where $\sigma_{k}=\alpha_{k}\in \Delta \left(S\right)$. Then,*

$$H_{n}\left(\sigma ,t\right)=\sum_{k=1}^{n}H\left(\alpha_{k}\right)$$

*and*

$$h\left(\sigma ,t\right)=\underset{k\to \infty }{lim sup}\frac{1}{n}\sum_{k=1}^{n}H\left(\alpha_{k}\right).$$

*Moreover,*

$$E\left(\sigma ,t\right)=\underset{M\to \infty }{lim sup}M\left(\frac{H(\alpha_{M})}{M}-\underset{k\to \infty }{lim sup}\frac{1}{n}\sum_{k=1}^{n}H\left(\alpha_{k}\right)\right).$$

*In addition, if $\alpha_{k}=\alpha$ for every k, then $H_{n}\left(\sigma ,t\right)=nH\left(\alpha \right)$ and*

h(σ,t)=H(α).

*Thus,*

E(σ,t)=0andE(σ)=0.

From Examples 2 and 3, we can see that excess *s*-entropy doesn’t respond to the unpredictability of a one-stage game. In both examples, a player plays in an unpredictable way (playing mixed strategy) but not in a complex way. Nevertheless, it detects unpredictability of a long-run play that is how predictable is the change in a player’s mixed strategy (probability distribution).

**Example** **4** (Periodic strategy). *Suppose that σ is a periodic strategy of period p. Then, h(σ,t)=0 and E(σ,t)=logp for any t∈T. Thus, E(σ)=logp.*

**Example 5** (Infinite excess*s*-entropy)**.** *Let $\sigma =(\sigma_{k})$ be such that $\sigma_{k}=\left(\frac{1}{2^{M}},\frac{2^{M}-1}{2^{M}}\right)$, where M∈N is such that $k\in [10^{M-1},10^{M})$. Then, h(σ,t)=0 and $E\left(\sigma ,t\right)=\sum_{k=1}^{\infty }\left(H_{n+1}\left(\sigma ,t\right)-H_{n}\left(\sigma ,t\right)\right)=\infty$. Therefore, E(σ)=∞.*

We can see that we should expect infinite excess *s*-entropy when changes of strategies are frequent, while, if changes are rare, the excess *s*-entropy should be finite. Now, we show another example which catches this intuition.

**Example** **6.** *Let us consider a two-player matrix game, where*

A={Top,Bottom},B={Left,Right}.

*The first player plays “Top” as long as Player 2 chooses “Left”. If Player 2 plays “Right” at the stage m for the first time, then Player 1 chooses a mixed action with distribution $\left(\frac{1}{2},\frac{1}{2}\right)$ (“Top” and “Bottom” are equally probable) for the following $m^{2}$ stages. After this time, he always chooses “Top”.* *Therefore, for every t∈T, the play $\left(X_{n}\right)$ induced by (σ,t) is*

$$X_{n}=\left(\mathrm{Top},\mathrm{Left}\right)\;\;\mathrm{for}\;\mathrm{every}\;\;n,$$

*or there exists m∈N such that*

$$X_{n}=\left\{\begin{array}{cc} (\mathrm{Top},\mathrm{Left}), & \mathrm{for}\;n=1,\ldots ,m-1\\ (\mathrm{Top},\mathrm{Right}), & \mathrm{for}\;n=m\\ (\left(\frac{1}{2},\frac{1}{2}\right),t_{n}), & \mathrm{for}\;n=m+1,\ldots ,m+m^{2}\\ (\mathrm{Top},t_{n}), & \mathrm{for}\;n\geq m+m^{2}+1. \end{array}\right.$$

*If the first condition is fulfilled, then $H_{n}\left(\sigma ,t\right)=0$ for every n and therefore E(σ,t)=0. If Player 2 plays “Right” at some stage (m), then*

$$H_{n}\left(\sigma ,t\right)=\left\{\begin{array}{cc} 0, & \mathrm{for}\;n=1,\ldots ,m\\ n-m, & \mathrm{for}\;n=m+1,\ldots ,m+m^{2}\\ m^{2}, & \mathrm{for}\;n\geq m+m^{2}+1. \end{array}\right.$$

*Therefore,*

h(σ,t)=0,

$$E\left(\sigma ,t\right)=m^{2},$$

*and*

$$E\left(\sigma \right)=m^{2}.$$

One may ask how this concept works and how this quantity can be calculated in more sophisticated and complex problems. Although this is not the aim of this article, we shortly discuss this here. The purpose of this section was to give illustrative examples, showing the intuition behind the introduced quantity. Nevertheless, to describe how excess *s*-entropy can be used in more complex games, we observe that we are building on the theory of entropy convergence rates. Therefore, we are able to use tools and ideas designed to study these rates. First, looking through the lens of information theory, Feldman et al. showed that excess entropy is the proper tool to detect and analyze patterns produced by a process [23,24]. It is able to distinguish between different patterns that have the same structure factors. However, E(σ,t) is the excess entropy of a process generated by (σ,t). Thus, excess *s*-entropy of a strategy σ with respect to a given strategy *t* can be used to detect and quantify patterns produced by a play (σ,t)(E(σ,t) can be calculated numerically). Second, from a dynamical systems perspective, we can study entropy convergence rates using different tools, especially from symbolic dynamics.

#### 2.4.3. Excess *s*-Entropy for Stationary Strategies

As we can see from the previous section, excess *s*-entropy agrees with the intuition of the complexity of the strategy. The natural question to ask is how we can bound excess *s*-entropy. Here, we will concentrate on stationary strategies. First, from Corollary 1, if a play induces stationary process, then

$$E\left(\sigma ,t\right)=\lim_{n\to \infty }n\left(\frac{1}{n}H_{n}\left(\sigma ,t\right)-h\left(\sigma ,t\right)\right).$$

In Theorems 2 and 3, we give few properties of the excess *s*-entropy.

**Theorem** **2.** *Let σ be a stationary strategy. Then, the following conditions are equivalent:*

*1*. *E(σ,t)=0,*
*2*. *$H_{n}\left(\sigma ,t\right)=nh\left(\sigma ,t\right)$ for all n∈N,*
*3*. *$H_{1}\left(\sigma ,t\right)=h\left(\sigma ,t\right)$.*

**Proof.**  We will prove equivalence of conditions showing implications (1)⇒(2)⇒(3)⇒(1).

To show implication from (1) to (2), it is sufficient to see that, because (by Lemma 3) the sequence $\left(g_{n}\left(\sigma ,t\right)\right)$ is decreasing, it has to be equal to h(σ,t) for every *t*. Condition (3) is an immediate consequence of (2). Noticing that by (3) we have $g_{0}\left(\sigma ,t\right)=H_{1}\left(\sigma ,t\right)$ and $\left(g_{n}\left(\sigma ,t\right)\right)$ decreasing implies (1). □

Moreover, for stationary strategies, we have a simple lower bound for excess *s*-entropy:

**Theorem** **3.** *If σ is stationary, then $h_{1}\left(\sigma ,t\right)\leq E\left(\sigma ,t\right)$ and*

$$E\left(\sigma \right)\geq \underset{t\in T}{\sup}h_{1}\left(\sigma ,t\right).$$

**Proof.**  First, let $G_{n}\left(\sigma ,t\right)=H_{n+1}\left(\sigma ,t\right)-H_{n}\left(\sigma ,t\right)$. Then,

$$H_{1}\left(\sigma ,t\right)-G_{1}\left(\sigma ,t\right)=G_{0}\left(\sigma ,t\right)-G_{1}\left(\sigma ,t\right)=\sum_{k=1}^{n-1}\left(G_{k-1}\left(\sigma ,t\right)-G_{k}\left(\sigma ,t\right)\right).$$

Converging with *n* to infinity results in

$$\begin{array}{c} h_{1}\left(\sigma ,t\right)=g_{0}\left(\sigma ,t\right)=H_{1}\left(\sigma ,t\right)-h\left(\sigma ,t\right)=\sum_{k=1}^{\infty }\left(G_{k-1}\left(\sigma ,t\right)-G_{k}\left(\sigma ,t\right)\right)\\ \leq \sum_{k=1}^{\infty }k\left(G_{k-1}\left(\sigma ,t\right)-G_{k}\left(\sigma ,t\right)\right)=\sum_{k=1}^{\infty }k\left(g_{k-1}\left(\sigma ,t\right)-g_{k}\left(\sigma ,t\right)\right)\leq E\left(\sigma ,t\right). \end{array}$$

 □

## 3. Discussion

In this article, we proposed a new measure of statistical complexity and unpredictability of a strategy—excess strategic entropy. Because of its entropy-based origin, we were able to catch intuition behind this quantity by understanding how entropy and entropy convergence rates work in the context of game theory. We showed and discussed its properties. To simplify discussion, we introduced this quantity for a two-player game, but it can be easily extended to games with more players. Lastly, in this section, we look at two possible applications of this quantity.

It is natural to restrict strategies of a player to those of finite statistical complexity. Obviously starting from the Nash equilibrium excess *s*-entropy (and complexity of a strategy) will be equal to zero, as the player won’t be eager to change his strategy. Nevertheless, if a player is not in the Nash equilibrium from the beginning, he may prefer to choose one of the mixed strategies which is not too complicated—for instance that doesn’t force him to change a one-shot strategy too much. Thus, restricting the set of strategies to those with bounded excess *s*-entropy seems to be a natural choice. Even if the player wants to “converge to the Nash equilibrium”, he may prefer those Nash equilibria to which his sequences of one-shot strategies converge using strategies with bounded excess *s*-entropy. Moreover, results obtained by Neyman et al. [7,25,26,27] suggest that restricting strategies to those of bounded strategic entropy can impact both Nash equilibria of the player and response of unrestricted players. Similarly to their considerations on strategies with small strategic entropy, restricting strategies to those with small excess *s*-entropy will impose high predictability of the mixed strategy (probability distribution which he uses is predictable), and will impact the possibility of choosing the best response strategy by the player with an unrestricted set of strategies. Therefore, bounded excess *s*-entropy strategies should be thoroughly explored.

Another research area where studying excess *s*-entropy may give a better insight are reputation models. The notion that commitment is valuable has long been a critical insight of non-cooperative game theory, and has deeply affected a number of social science fields, including macroeconomics, international finance, and industrial organization. It seems natural to expect that agents playing in the long-run competition will base their decisions on the reputation of the opponent. Moreover, if the opponent is more predictable (in the sense that the probability distribution which he uses is predictable), then the player can find a better response to the predicted strategy, or may prefer to play with the more predictable player. It should be added that using entropy-based measures in reputation models is not a new idea. For instance, in [28], Ekmekci et al. used entropy bounds to study the impact of unobservable stochastic replacements for the long-run player in the classical reputation model with a long-run player and a series of short-run players. Other applications of entropy concept to reputation models were made, e.g., in [29,30].

## 4. Materials and Methods

Methods used in article are standard methods and are thoroughly discussed in Section 2. 
